# Optimization of Cannula Visibility during Ultrasound-Guided Subclavian Vein Catheterization, via a Longitudinal Approach, by Implementing Echogenic Technology

**DOI:** 10.1155/2012/617149

**Published:** 2012-04-23

**Authors:** Konstantinos Stefanidis, Mariantina Fragou, Nicos Pentilas, Gregorios Kouraklis, Serafim Nanas, Richard H. Savel, Ariel L. Shiloh, Michel Slama, Dimitrios Karakitsos

**Affiliations:** ^1^Radiology Department, Evangelismos University Hospital, 10676 Athens, Greece; ^2^Intensive Care Unit, General State Hospital of Athens, 11523 Athens, Greece; ^3^Second Department of Propedeutic Surgery, Faculty of Medicine, National and Kapodistrian University of Athens, and Laiko General Hospital, 11527 Athens, Greece; ^4^1st Critical Care Department, Evangelismos University Hospital, 10676 Athens, Greece; ^5^Jay B. Langner Critical Care Service, Department of Medicine, Montefiore Medical Center, Albert Einstein College of Medicine, Bronx, NY 10461, USA; ^6^Intensive Care Unit, CHU Sud Amiens, 80054 Paris Cedex 1, France; ^7^Unité INSERM 1088, University Picardie Jules Vernes, Amiens, 80025 Paris, France

## Abstract

*Objective*. One limitation of ultrasound-guided vascular access is the technical challenge of visualizing the cannula during insertion into the vessel. We hypothesized that the use of an echogenic vascular cannula (EC) would improve visualization when compared with a nonechogenic vascular cannula (NEC) during real-time ultrasound-guided subclavian vein (SCV) cannulation in the ICU. *Material and Methods*. Eighty mechanically ventilated patients were prospectively enrolled in a randomized study that was conducted in a medical-surgical ICU. Forty patients underwent EC and 40 patients were randomized to NEC. The procedure was ultrasound-guided SCV cannulation via the infraclavicular approach on the longitudinal axis. *Results*. The EC group exhibited increased cannula visibility as compared to the NEC group (92%±3% versus 85 ± 7%, resp., *P* < 0.01). There was strong agreement between the procedure operators and independent observers (*k* = 0.9, 95% confidence intervals assessed by bootstrap analysis = 0.87 to 0.93; *P* < 0.01). Access time (12.1 s ± 6.5 versus 18.9 s ± 10.9) and the perceived technical difficulty of the ultrasound method (4.5 ± 1.5 versus 7.5 ± 1.5) were both decreased in the EC group compared to the NEC group (*P* < 0.05). *Conclusions*. Echogenic technology significantly improved cannula visibility and decreased access time and technical complexity optimizing thus real-time ultrasound-guided SCV cannulation via a longitudinal approach.

## 1. Introduction

Real-time ultrasound-guided central venous cannulation has been associated with higher success rates, faster access times, and a reduction in mechanical complications, when compared to landmark techniques [1–6]. Mechanical complications occur more frequently when accessing the subclavian vein (SCV) compared to the other sites of central venous access [6–8]. We recently demonstrated that ultrasound-guided SCV cannulation, while technically demanding, was superior to landmark methods in a cohort of intensive care unit (ICU) patients [6]. Our ultrasound method was based on the implementation of a step-by-step guided technique [6]. Cannula visualization is fundamental to the safety and efficacy of all ultrasound-guided methods, but no single technology meant to improve cannula echogenicity has been widely adopted [9–14]. The value of this technology has not been formally studied in the ICU setting [9, 11, 12, 15]. Recently, a vascular cannula (VascularSono, Pajunk, GmbH, Medizintechnologie, Geisingen, GermanyVascularSono) incorporating “Cornerstone” reflectors on the distal 2 cm, to increase echogenicity, was developed based on technology previously used in regional anesthesia needles [16]. We hypothesized that the use of an echogenic vascular cannula (EC) would improve visualization when compared with a nonechogenic vascular cannula (NEC) (Arrow Howes, PA, U.S.A) during real-time ultrasound-guided SCV cannulation in the ICU.

## 2. Materials and Methods

During 2011, eighty patients who required central venous access were prospectively enrolled in this randomized study that was conducted in a medical-surgical ICU. Forty patients underwent EC and 40 patients were randomized to NEC. The procedure was ultrasound-guided SCV cannulation via the infraclavicular approach on the longitudinal axis. All patients were sedated and mechanically ventilated. Randomization was performed by means of a computer-generated random-numbers table, and patients were stratified with regards to age, gender, and body mass index (BMI). Block randomization was used to ensure equal numbers of patients in the above groups [3]. All physicians who performed the procedures had at least five years of experience in central venous catheter placement. The study was approved by the institutional ethics committee, and appropriate informed consent was obtained. 

Chest radiography was used to assess catheter placement after the procedure, as previously described [6, 17]. Mechanical complications were defined as arterial puncture, hematoma, hemothorax, pneumothorax, injury to the brachial plexus as well as to the phrenic nerve, catheter misplacement, and cardiac tamponade [6].

### 2.1. Real-Time Ultrasound-Guided SCV Cannulation

**Figure 1:**
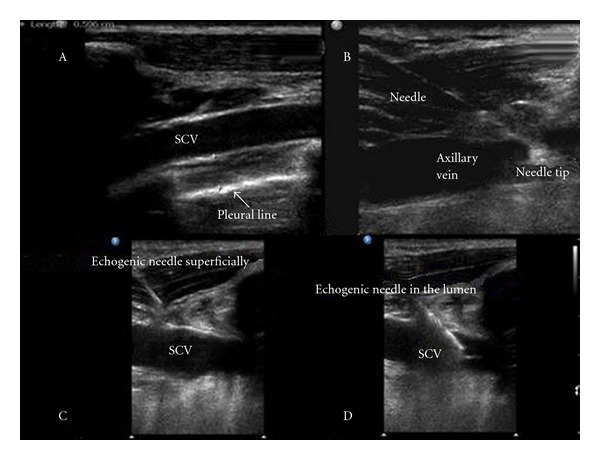
The subclavian vein (SCV) scanned just above the pleural line (A); axillary vein cannulation by nonechogenic cannula on the longitudinal axis (B); snapshots of SCV cannulation by echogenic cannula depicting its tip superficially (C) and in the vessel's lumen (D), respectively.

**Figure 2:**
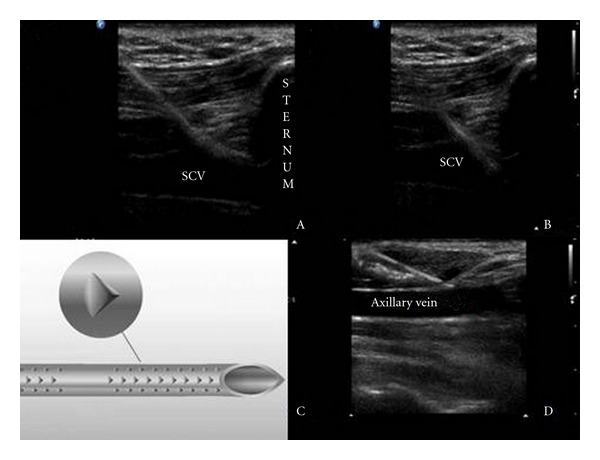
Echogenic cannula entering the SCV just adjacent to the sternum (A B); the former incorporates “Cornerstone” reflectors mainly arranged at its distal 2 cm (C), which increase significantly its visibility (D).

All patients were placed in Trendelenburg position and were cannulated as described in detail by Fragou et al. [6]. Triple-lumen catheters were used in all cases and all procedures were performed under controlled and nonemergent conditions in the ICU. Standard sterile precautions were utilized. The EC and NEC were both 18 gauge cannulas specifically intended for use in vascular access. Ultrasonography was performed with an HD11 XE ultrasound machine (Philips, Andover, MA, USA) equipped with a high-resolution 7.5–12 MHz transducer, which was covered with sterile ultrasonic gel and wrapped in a sterile sheath (Microtec medical intraoperative probe cover, 12 cm × 244 cm). Using the infraclavicular approach, on the longitudinal axis, sonoanatomic landmarks (such as the acoustic shadows of the underlying first thoracic rib and of the sternum) were identified, as well as, the axillary and SCV vein (Figures 1 and 2). Doppler techniques were utilized to confirm the two-dimensional (2D) findings. Vessels were cannulated using the Seldinger technique under real-time ultrasound guidance.

### 2.2. Data Acquisition, Study Protocol, and Outcome Measures

The cannulation was performed by a single operator and was observed by a second physician. The operators and observers were blinded to the cannula used. Following each procedure, the operator and the observer were asked to score the percentage of time they were able to continuously visualize the cannula; a 10-point scale was used (ranging from 1 equals 0%–10%, to 10 equals 90%–100%). Operators were asked to rate the perceived technical difficulty and complexity of the task also using a 10-point scale, in which 0 was most simple and 10 was most complex [6]. The observer measured access time, number of attempts, and complications. Access time was defined as the time between penetration of skin and aspiration of venous blood. 

Data was collected using a standardized form and was entered in a database. We documented baseline patient characteristics, side of catheterization, presence of risk factors for difficult venous cannulation, previous difficulties during cannulation, previous mechanical complications, known vascular abnormalities, and untreated coagulopathy (international normalization ratio > 2; activated partial thromboplastin time > 1.5; platelets < 50 × 10^9^ litre^−1^).

### 2.3. Statistical Analysis

Data were expressed as mean ± standard deviation (SD). Student's *t*-test for independent means, *χ* ^2^ analysis, or Fisher's exact test where appropriate were used to identify differences between the two groups. A *P*  value (twosided in all tests) of <0.05 was considered significant. Study power was based on data from a previous needle visibility study and was adjusted for our intervention [18]. Assuming data to be nonparametric, power sample analysis gave a minimum sample size of 40 cannulations. Wilcoxon rank-sum test was used to compare tip visibility data for the 2 groups. The agreement between the operator and the observer cannula visibility results was evaluated by Cohen's weighted kappa, while 2.5th and 97.5th percentiles of 5,000 bootstrap replicates estimated 95% confidence intervals. The bootstrap is a resampling method used for estimating a distribution, from which various measures of interest can be calculated [19]. Statistical analysis was performed using SPSS software, version 11.0 (SPSS Inc. Chicago, IL, USA).

## 3. Results

Baseline characteristics of the study population are presented in Table 1. There were no significant differences in age, gender, body mass index (BMI), and presence of risk factors for difficult venous cannulation between the NEC and the EC groups. No cases of preexisting thrombosis were identified. 

**Figure 3:**
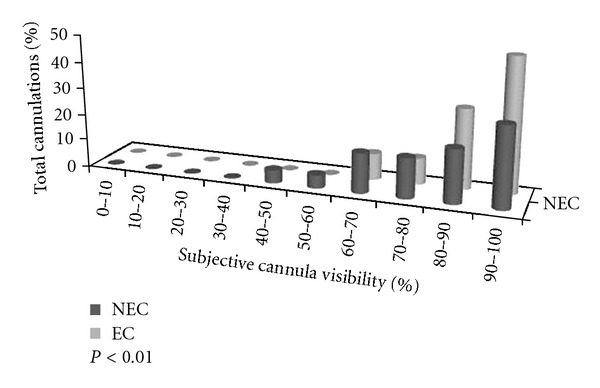
Subjective percentage of cannula visibility assessments (echogenic cannula, EC: gray; nonechogenic cannula, NEC: black).

Results of cannula visibility are presented in Figure 3. Operators reported improved cannula visualization in the EC group when compared to the NEC group (92% ± 3% versus 85 ± 7%, respectively; *P* < 0.01). The agreement between the operators and observers was statistically significant (*k*  equal  0.9, 95% confidence intervals assessed by bootstrap analysis = 0.87–0.93; *P* < 0.01). 

Results of the secondary outcomes are presented in Table 2. There were no statistically significant differences noted in mechanical complications between the two groups. Access time (12.1 s ± 6.5 versus 18.9 s ± 10.9) and the perceived technical difficulty of the procedure (4.5 ± 1.5 versus 7.5 ± 1.5) were both decreased in the EC group compared to the NEC group (*P* < 0.05). Examples of cannula visibility of EC and NEC during ultrasound-guided SCV and axillary vein cannulation, on the longitudinal axis, are shown in Figures 1 and 2.

## 4. Discussion

 Our study demonstrated improved cannula visibility with the use of EC during ultrasound-guided SCV cannulation. The likelihood of visualizing the cannula during a longitudinal approach is already reasonably high [6]; nevertheless, the use of EC statistically increased the likelihood of continued successful cannula visualization. In addition, the utilization of EC resulted in significantly reduced access times and perception of technical difficulty. EC represents a brightly echogenic vascular puncture cannula which incorporates “Cornerstone” reflectors mainly arranged at the distal 2 cm of the needle. These reflectors guarantee the visibility of the cannula shaft, independent of the puncture angle according to the manufacturer. The principle is the same as that used in bicycle reflectors, where light is reflected back to its source regardless of the angle at which it approaches [15–17]. The present results suggested that the echogenic technology significantly improved cannula visibility during real-time ultrasound-guided SCV cannulation. Our methodology was designed to test EC in actual clinical practice in the ICU, where image acquisition is affected or limited by the presence of various factors such as obesity, subcutaneous air, edema, trauma and mechanical ventilation [1–8]. The use of EC may improve image acquisition and success rates in technically challenging cases of vascular access.

There is no definitive method for objective assessment of cannula visibility. Previous studies used scoring systems with skilled observers rating static images [9–14]. Other groups have suggested objective measures of cannula visibility in still images [15, 18]. We aimed to examine cannula visibility during central venous cannulation, under real-time clinical conditions.

Although interpretation of dynamic 2D ultrasound images remains subjective, we used an analytical 10-point scale, along with a “dual” evaluation model of operators and observers. We demonstrated that, high operator and observer agreement existed between the subjective estimations of cannula visibility rates. 

The study has several limitations. Despite the fact that the operators were blinded at the initiation of the procedure, the two vascular cannulas inherently exhibited different ultrasonographic appearance and could possibly be differentiated. The dimensions of the Cornerstone reflectors are determined by the frequency of the ultrasound with which they are designed to work. Lower frequencies may require broader dimensions, but these are limited by the wall thickness of the cannula [9–15] In this study, the echogenic cannulas used were specifically designed for central venous access. 

We failed to find any significant reduction of mechanical complications. This may be due to the fact that an in-plane technique was used in all cases, moreover our baseline mechanical complication rate was extremely low (given the fact that our study group is highly skilled in ultrasound-guided vascular access), and that the sample size was rather small. Finally, let us underline that complications exist even with ultrasound-guidance (i.e., hematoma resulting from inadvertent arterial damage either to the adjacent main artery or some of the many branches in this area) during SCV cannulation [6, 20]. 

In conclusion, our investigation demonstrated that the use of EC significantly improved THE cannula visibility and decreased the vascular access time as well as perceived technical complexity during real-time ultrasound-guided SCV cannulation. Our data provide clinical rationale to study the evolving field of enhanced echogenic ultrasound technology. Further studies are required to determine if EC is cost-effective and changes overall outcomes in the ICU.

## Figures and Tables

**Table 1:** Baseline characteristics of the study population; values are presented either in percentages or as mean ± SD.

Characteristics	EC group (*n* = 40)	NEC group (*n* = 40)
Age (years)	50 ± 10.5	51 ± 9.9
Gender (male/female ratio)	0.51 ± 0.4	0.52 ± 0.5
APACHE II score	20.2 ± 3.1	20.3 ± 3.3
Diagnosis upon admission		
Trauma without brain injury	5 (12.5%)	7 (17.5%)
Trauma with brain injury	15 (37.5%)	11 (27.5%)
Burn	2 (5%)	3 (7.5%)
ARDS	3 (7.5%)	5 (12.5%)
Sepsis	5 (12.5%)	7 (17.5%)
Postsurgical complications	10 (25%)	7 (17.5%)
Side of catheterization (left/right)	19/21	18/22
Body mass index (kg/m^2^)	22.9 ± 5.1	23.8 ± 4.2
Prior catheterization	10 (25%)	10 (25%)
Limited sites for access attempts	3 (7.5%)	3 (7.5%)
Previous difficulties during	5 (12.5%)	5 (12.5%)
Catheterization		
Previous mechanical complications	2 (5%)	2 (5%)
Known vascular abnormality	1 (2.5%)	1 (2.5%)
Untreated coagulopathy	0 (0%)	1 (2.5%)
Skeletal deformity	1 (2.5%)	0 (0%)

APACHE II score: acute physiology and chronic health evaluation score II; ARDS: acute respiratory distress syndrome; NEC: nonechogenic cannula, EC: echogenic cannula.

**Table 2:** Secondary outcome measures in the EC group versus the NEC group.

Outcome measures	EC group (*n* = 40)	NEC group (*n* = 40)
Access time (sec)	12.1 ± 6.5 (5.5–20.4)*	18.9 ± 10.9 (9.5–29.4)
Success rate (%)	40 (100%)	40 (100%)
Average number of attempts artery puncture	1 ± 0.3 (1–1.5) 0 (0%)	1.1 ± 0.5 (1–1.8) 1 (2.5%)
Hematoma	0 (0%)	1 (2.5%)
Pneumothorax	0 (0%)	0 (0%)
Hemothorax	0 (0%)	0 (0%)
Catheter misplacement	0 (0%)	0 (0%)
Damage of the brachial plexus	0 (0%)	0 (0%)
Phrenic nerve injury	0 (0%)	0 (0%)
Technical difficulty (scale 1 to 10)	4.5 ± 1.5*	7.5 ± 1.5

EC: echogenic cannula, NEC: nonechogenic cannula; Comparisons between the NEC and the EC group of patients; *P* < 0.05*; access time and average number of attempts are expressed as mean ±  SD (95% confidence intervals).
